# Posttreatment Motivation and Alcohol Treatment Outcome 9 Months Later: Findings From Structural Equation Modeling

**DOI:** 10.1037/a0037981

**Published:** 2014-09-22

**Authors:** Sarah Cook, Nick Heather, Jim McCambridge

**Affiliations:** 1 London School of Hygiene & Tropical Medicine; 2 Department of Psychology, Faculty of Health and Life Sciences, Northumbria University; 3 London School of Hygiene & Tropical Medicine

**Keywords:** alcohol problems, treatment, readiness to change, motivation, outcome predictors

## Abstract

***Objective:*** To investigate the association between posttreatment motivation to change as measured by the Readiness to Change Questionnaire Treatment Version and drinking outcomes 9 months after the conclusion of treatment for alcohol problems. ***Method:*** Data from 392 participants in the United Kingdom Alcohol Treatment Trial were used to fit structural equation models investigating relationships between motivation to change pre- and posttreatment and 5 outcomes 9 months later. The models included pathways through changes in drinking behavior during treatment and adjustment for sociodemographic information. ***Results:*** Greater posttreatment motivation (being in action vs. preaction) was associated with 3 times higher odds of the most stringent definition of positive outcome (being abstinent or entirely a nonproblem drinker) 9 months later (odds ratio = 3.10, 95% confidence interval [1.83, 5.25]). A smaller indirect effect of pretreatment motivation on this outcome was seen from pathways through drinking behavior during treatment and posttreatment motivation (probit coefficient = 0.08, 95% confidence interval [0.03, 0.14]). A similar pattern of results was seen for other outcomes evaluated. ***Conclusion:*** Posttreatment motivation to change has hitherto been little studied and is identified here as a clearly important predictor of longer term treatment outcome.

## Abstract (public health significance statement toc summary)

This study found that those individuals who reported that they were ready to change their drinking at the end of a treatment program were much more likely to show positive outcomes 9 months subsequently than were persons not indicating such a readiness to change. This suggests that attempting to enhance motivation throughout the process may be an important component of successful alcohol treatment.

Identifying how motivation to change is related to positive outcomes is important for understanding how treatment for alcohol or other behavioral problems can work. Pretreatment stage of change has been found to be an important predictor of outcome for a wide range of disorders (Norcross, Krebs, & Prochaska, 2011), including several aspects of treatment for alcohol problems (Connors et al., 2000; Hernandez-Avila, Burleson, & Kranzler, 1998; Isenhart, 1997; Project MATCH Research Group, 1998).

Three studies have previously investigated three different motivational measures, all based on stages of change, assessed at the conclusion of treatment for alcohol problems. A profile analysis generated from stage of change variables in Project MATCH identified a relationship between more strongly endorsing action posttreatment, measured using the University of Rhode Island Change Assessment (DiClemente & Hughes, 1990), and longer term abstinence (Carbonari & DiClemente, 2000). Another analysis of data from two cognitive behavior therapy alcohol treatments for women found posttreatment motivation, as measured by the Stages of Change Readiness and Treatment Eagerness Scale (Miller & Tonigan, 1996), was a mediator of the relationship between social support for drinking and drinking frequency 6 months later (Hunter-Reel, McCrady, Hildebrandt, & Epstein, 2010).

A third study that was based on data from the United Kingdom Alcohol Treatment Trial (UKATT; UKATT Research Team, 2005) found that posttreatment, but not pretreatment, stage of change, measured by the Readiness to Change Questionnaire Treatment Version (Heather & Hönekopp, 2008), was predictive of drinking outcomes at follow-up 9 months after treatment ended (Heather & McCambridge, 2013). The associations in this study were greatly reduced after adjusting for drinking behavior during treatment: Effect sizes were smaller and did not obtain statistical significance on the most stringent definitions of positive outcome (Heather & McCambridge, 2013). However, motivation to address alcohol problems will be highly interconnected with drinking behaviors before, during, and after treatment, making study of their effects complex (Rollnick, 1998).

The conceptual framework guiding the present study posits that interconnected pathways between variables are structured by time and that mediator and moderator variables may have proximal or distal impacts on one another, the strength of which may vary with time. Structural equation modeling is a flexible statistical technique that can be used to analyze interconnected pathways between variables and thus provide more detailed information on their relationships throughout the treatment process. Our primary hypothesis was that motivation to change drinking posttreatment predicts outcome of treatment for alcohol problems. Our aim in this study was to use structural equation modeling to further investigate the associations previously observed in the UKATT data between posttreatment motivation to change drinking and drinking outcomes roughly 9 months after the conclusion of treatment (Heather & McCambridge, 2013), including delineation of pathways through changes in drinking behaviors, paying careful attention to temporal sequencing in the context of an explicitly longitudinal perspective on change.

## Method

### Study Sample and Design

The UKATT (UKATT Research Group, 2005) was a multicenter randomized controlled trial carried out in five treatment centers in the United Kingdom that compared two different treatments for alcohol problems: motivational enhancement therapy (MET) and social behavior and network therapy (SBNT). This was a pragmatic trial and the study population comprised clients who would normally receive an offer of treatment for alcohol problems in publicly funded treatment services in the United Kingdom. No differences were found between the two treatment groups on any of the drinking outcomes (UKATT Research Team, 2005). Motivation to change and drinking behaviors were measured pretreatment and then at 3 months (when all treatment was ended) and 9 months later, that is, 12 months after entry to the trial. UKATT recruited 742 clients (MET = 422, SBNT = 320) attending treatment voluntarily. Because our research question was related to treatment process, only clients who attended at least one session were included (*n* = 590). We also examined those with complete data available on the variables of interest at all three time points because the aim of this study was to model the interrelationship between these variables over time. This resulted in a sample of 392 clients included for the present study. There were some differences between this subsample and those who were not included in terms of education (those included were more likely to have been educated to degree level or equivalent [12.2% vs. 7.4%, *p* = .036] and less likely to have no educational qualification [30.4% vs. 41.7%, *p* = .002]). The included subsample also had somewhat less severe problems at baseline (lower mean scores on the Leeds Dependence Questionnaire [15.1 vs. 16.4, *p* = .030] and Alcohol Problems Questionnaire [10.4 vs. 11.7, *p* < .001]).

### Measures

Outcome variables were derived from Form 90 data on alcohol consumption in the past 90 days (Miller, 1996) and the Alcohol Problems Questionnaire (APQ; Drummond, 1990). Data from the Form 90 and APQ were combined to derive three binary treatment outcome variables based on a composite categorical variable developed by Heather and Tebbutt (1989):

Outcome 1: Abstinent or nonproblem drinker (no alcohol consumption in the past 90 days or drinking with a score of zero on the APQ indicating no evidence of any alcohol problems)
Outcome 2: At least much improved (abstinent or drinking with a reduction in APQ score from baseline to follow up of at least two thirds)
Outcome 3: At least somewhat improved (abstinent or drinking with a reduction in APQ score from baseline of at least one third)

These outcomes are principally concerned with the resolution of alcohol problems and vary in the stringency of the definition of a positive outcome. The additional outcomes investigated were two continuous measures of drinking behavior derived from the Form 90 data:

Outcome 4: Drinks per drinking day (DDD) in the past 90 days, with abstinent clients given a score of zero
Outcome 5: Percentage of days abstinent (PDA)

These were the same outcome measures used by Heather and McCambridge (2013).

Motivation to change was assessed using the revised edition of the Readiness to Change Questionnaire Treatment Version, which is designed for use in alcohol-treatment seeking populations (Heather & Hönekopp, 2008) and which refers to both quitting and cutting down on alcohol consumption. This 12-item instrument was used to calculate scores on three stages of change: precontemplation, contemplation, and action. Clients are assigned a stage of change based on the scale on which they score highest, with ties being decided in favor of the stage farthest along the cycle of change. As no clients were in the precontemplation stage at pretreatment and only three were at posttreatment, we defined actively changing drinking (action stage) versus not actively changing drinking (precontemplation + contemplation stages = preaction) as a binary variable.

Sociodemographic variables measured pretreatment were age (coded into 5-year groups), education (coded as no qualifications, some qualifications, and degree or equivalent qualifications), and marital status (married and/or cohabiting or not). Pretreatment score on the Leeds Dependence Questionnaire (Raistrick et al., 1994) assessing the severity of dependence at treatment entry was also included in the model as a predictor of drinking behavior during treatment.

### Statistical Analyses

**Figure 1 fig1:**
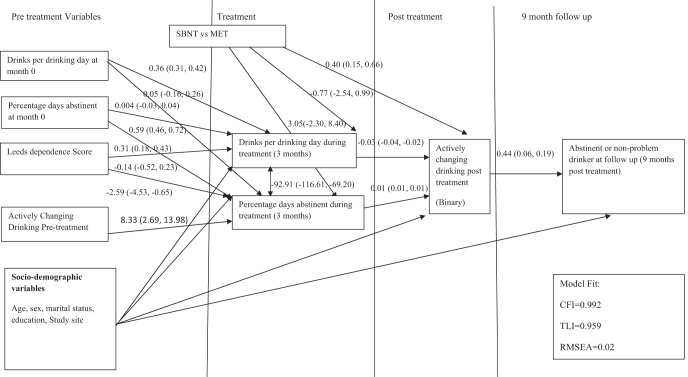
Structural equation model examining relationship between actively changing drinking following treatment and long term drinking outcomes. *N* = 392. Coefficients are linear regression coefficients for continuous outcomes and probit coefficients for binary outcomes. SBNT = social behavior and network therapy; MET = motivational enhancement therapy; CFI = comparative fit index; TLI = Tucker–Lewis index; RMSEA = root-mean-square error of approximation.

The relationship between motivation to change (pre- and posttreatment) and treatment outcomes 9 months posttreatment was assessed using the structural equation model shown in Figure 1 for Outcome 1. This model was specified a priori by considering the likely temporal relationship between variables. For example, effects of pretreatment motivation to change on drinking outcomes at 9 months posttreatment were considered to be through effects on intermediate variables (drinking during treatment and posttreatment motivation to change). This hypothesis was tested by adding in a direct effect of pretreatment motivation to change on treatment outcomes at 9 months in a sensitivity analysis.[Fig-anchor fig1]

The model is divided by time into four sections—pretreatment, within treatment, posttreatment, and 9 months follow-up—to help elucidate the interrelationships between variables over time. Being abstinent or a nonproblem drinker (Outcome 1) was identified a priori as the main treatment outcome of interest, with models also fitted for the other two binary outcomes comprising less stringent definitions of positive outcome and the continuous outcomes (PDA and DDD) at 9 months posttreatment. Models were fitted separately for each outcome but with the same specified associations between the pre- and posttreatment variables because there was no reason to believe these relationships would differ between treatment outcomes. All models were adjusted for potential confounding by sociodemographic variables (age, sex, education, and marital status). Study site was also included as a confounder as this could represent both differences related to treatment services and geographic location.

Models were estimated using weighted least squares with mean and variance adjusted (WLSMV) but with maximum-likelihood estimation used to calculate odds ratios for the direct effects of posttreatment stage of change on binary drinking outcomes at 12 months. Model fit was assessed using the comparative fit index (CFI), Tucker–Lewis index (TLI), and the root-mean-square error of approximation (RMSEA). CFI and TLI values greater than .95 indicate good model fit, with a minimum of .90 indicating acceptable fit (Streiner, 2006; Tabachnik & Fidell, 1996). For the RMSEA, values greater than 0.10 indicate a bad fit, whereas values less than 0.08 indicate a reasonable fit and less than 0.05 indicate a good fit (Streiner, 2006).

## Results

The study sample included 392 clients (74.7% male). Mean age was 42.2 years (*SD* 9.9). 46 clients were nonproblem drinkers at 9 months follow up, and 55 were abstinent. Overall, 153/392 clients overall met the criteria for being much improved and 225/392 were at least somewhat improved.

The results for the most stringent definition of positive treatment outcome (Outcome 1, abstinent/nonproblem drinker at 9 months) are shown in Figure 1. Model fit was very good. Greater posttreatment motivation (being in action vs. preaction) was associated with 3.10 (95% CI [1.83, 5.25]) higher odds (equivalent probit coefficient = 0.44, 95% CI [0.29, 0.59]) of positive outcome at 9 months. There was also good evidence for an indirect effect of pretreatment motivation on being abstinent or a nonproblem drinker at 9 months via effects on DDD and PDA at 3 months and posttreatment motivation (probit coefficient = 0.08, 95% CI [0.03, 0.14]). This was not reduced by including a direct effect of pretreatment motivation on treatment outcome in the model. There was no evidence for a direct effect of pretreatment motivation (probit coefficient = −0.19, 95% CI [−0.10, 0.48]).

The same pattern of results was seen for Outcome 2 (at least much improved; odds ratio for posttreatment motivation = 2.84, 95% CI [1.85, 4.38], and probit coefficient for indirect effect of pretreatment motivation = 0.09, 95% CI [0.03, 0.16]) and for Outcome 3 (at least somewhat improved; odds ratio for posttreatment motivation = 3.27, 95% CI [2.21, 4.84], and probit coefficient for indirect effect of pretreatment motivation = 0.11, 95% CI [0.04, 0.18]). Model fit for Outcomes 2 and 3 was reasonable (for Outcome 2, CFI = .95, TLI = .72, RMSEA = 0.06; for Outcome 3, CFI = .93, TLI = .60, RMSEA = 0.08).

Findings were also similar for Outcomes 4 and 5. Drinks per drinking day at 9 months were 4.14 (95% CI [3.45, 4.82]) fewer in those in action versus preaction posttreatment and 0.93 (95% CI 0.31, 1.55) drinks fewer in those in action versus preaction pretreatment. Those in action versus preaction posttreatment had 12.03% (95% CI [9.11, 14.95]) more abstinent days during Months 10–12. Those in action versus preaction at the beginning of treatment had 3.19% (95% CI [0.86, 5.52]) more abstinent days. Models for continuous outcomes had poorer model fit (for DDD, CFI = 0.64, TLI = −0.88, RMSEA = 0.19; for PDA, CFI = .83, TLI = .12, RMSEA = 0.12).

There was no evidence for Outcomes 2–5 of any direct effects of pretreatment motivation to change. Estimates for indirect effects of pretreatment motivation to change on treatment outcome did not substantively change by adding in a direct effect to the model for any of the treatment outcomes.

In contrast to all previous UKATT findings, there was some evidence (*p* < .05) of a treatment effect: Those who received SBNT were more likely than those in the MET group to be actively trying to change their drinking at the end of treatment for three out of five of the treatment outcomes (Outcomes 1, 2, and 5).

## Discussion

Motivation to change, comparing those in action versus preaction at the conclusion of treatment for alcohol problems, was strongly associated with being abstinent or a nonproblem drinker at follow-up 9 months after treatment ended, approximately trebling the odds of this outcome. Pretreatment motivation had a lesser but nonetheless statistically significant indirect effect via effects on drinking behavior during treatment and posttreatment motivation. The same pattern of results was found for all other longer term treatment outcomes. These results, using a more sophisticated modeling approach, support and extend previous analyses of the same data set (Heather & McCambridge, 2013) by producing a more precise and indeed larger estimate of the effect of posttreatment motivation. Unlike the previous study, our study reveals an indirect effect of pretreatment motivation under the assumptions of no unmeasured confounders (Muthèn, 2011; VanderWeele, 2012) and no direct paths from the measured pretreatment variables to the outcomes, which shows the importance of considering change over time. Using a structural equation modeling approach enabled us to estimate more realistically the relationships between drinking behavior and motivation to change throughout the entire study period, taking account of the temporal nature of likely associations. These data add to the meager literature, comprising only two other treatment cohorts, for which different motivational measures were used.

This study used a binary motivational measure because almost all clients providing data at all three time points were in either the contemplation or the action stage of change, both pre- and posttreatment, and therefore there seemed little added benefit in using a more complex measure. The subsample used in this study had slightly less severe alcohol problems than did the overall UKATT study sample, which was broadly representative of the U.K. treatment population at the time the study was undertaken (Heather & McCambridge, 2013; UKATT Research Team, 2005), with implications for the generalizability of these data. Although measured motivation at treatment entry was similar among members of this group and the group not included in this study, the need to include those who attended at least one treatment session and also provided follow-up data posttreatment and at 12 months may mean there was differential loss to follow-up by motivation postrandomization, although it is difficult to assess this. Using a binary measure of motivation and restricting analyses to a subgroup of the UKATT population thus entails restrictions on the capacity to make inferences about the entire treatment population. In addition, although we have used here the Readiness to Change Questionnaire to measure motivation, there are different constructs and measures of motivation, including those not based on the stages of change (see Gaume, Bertholet, Daeppen, & Gmel, 2013). There is, therefore, a need for replication of analyses using different measures of motivation to fully understand motivation’s importance in treatment for alcohol problems.

These findings describe how drinking behavior changes over time and, notwithstanding that temporal sequencing rules out reverse causality, we make no direct causal inferences from these data given the observational nature of this study. Drinking measures for the 90 days prior to treatment (PDA and DDD) predict these same measures for the period during treatment. Pretreatment motivation also strongly predicts both of these measures during treatment. Reducing drinking is then associated with posttreatment motivation, which, in turn, predicts outcome 9 months later. If there is an underlying causal chain, capitalizing on motivation at the beginning of treatment and making progress during treatment thus appears important to longer term outcome, as is how treatment ends for clients and specifically their motivation to change their drinking at that point.

There was somewhat consistent evidence of a small treatment effect on posttreatment motivation favoring SBNT over MET. This counterintuitive finding could be explained if increased social support for change elicited by SBNT is more effective in motivating change efforts by the client than the mainly psychological processes targeted by MET. However, our finding contrasts with the previously reported analyses of UKATT outcomes, including no differences in the proportion of clients in the action stage of change posttreatment by treatment group (Heather & McCambridge, 2013). The reasons for these differences are not clear and further investigation is warranted.

Posttreatment motivation has been found to be a mediator of the relationship between baseline social support and drinking outcomes (Hunter-Reel et al., 2010). However, as far as we are aware, formal analyses of the role of motivation as a possible mediator of treatment effects has not been undertaken in alcohol treatment studies. In a related area, a brief motivational intervention was found to be more effective in decreasing negative drinking consequences through changes in motivation among emergency department attendees with injuries only in those who were already motivated to change before intervention (Stein et al., 2009). Further process studies are needed to test hypotheses about mediation and moderation of the effects of treatment for alcohol problems.

Korcha, Polcin, Bond, Lapp, and Galloway (2011) have drawn attention to the surprising absence of a longitudinal perspective on motivation in almost all existing alcohol and drug research, despite studies showing its importance for other behaviors such as smoking cessation (e.g., Boardman, Catley, Mayo, & Ahluwalia, 2005). Although it is possible that the lack of prior published studies may, to some degree, reflect publication bias, with null findings not reported, it is clear that posttreatment motivation is a neglected target for study in relation to treatment outcome. Further investigation of this somewhat novel candidate for mechanisms of behavior change and the application of a longitudinal perspective more broadly have potential for deepening the understanding of how alcohol treatment works.
